# Polyuria and cerebral vasospasm after aneurysmal subarachnoid hemorrhage

**DOI:** 10.1186/s12883-015-0446-6

**Published:** 2015-10-13

**Authors:** Robert J. Brown, Brian P. Epling, Ilene Staff, Gilbert Fortunato, James J. Grady, Louise D. McCullough

**Affiliations:** Department of Surgery, Division of Critical Care, Hartford Hospital, 80 Seymour Street, Hartford, 06102 USA; Department of Neurology, University of Connecticut Medical Center, 263 Farmington Avenue, Farmington, 06030 USA; University of Connecticut School of Medicine, 263 Farmington Avenue, Farmington, 06030 USA; Department of Research, Hartford Hospital, 80 Seymour Street, Hartford, 06102 USA

**Keywords:** Subarachnoid hemorrhage, Delayed cerebral ischemia, Vasospasm, Polyuria, Natriuresis

## Abstract

**Background:**

Natriuresis with polyuria is common after aneurysmal subarachnoid hemorrhage (aSAH). Previous studies have shown an increased risk of symptomatic cerebral vasospasm or delayed cerebral ischemia (DCI) in patients with hyponatremia and/or the cerebral salt wasting syndrome (CSW). However, natriuresis may occur in the absence of hyponatremia or hypovolemia and it is not known whether the increase in DCI in patients with CSW is secondary to a concomitant hypovolemia or because the physiology that predisposes to natriuretic peptide release also predisposes to cerebral vasospasm. Therefore, we investigated whether polyuria per se was associated with vasospasm and whether a temporal relationship existed.

**Methods:**

A retrospective review of patients with aSAH was performed. Exclusion criteria were admission more than 48 h after aneurysmal rupture, death within 5 days, and the development of diabetes insipidus or acute renal failure. Polyuria was defined as >6 liters of urine in a 24 h period. Vasospasm was defined as a mean velocity > 120 m/s on Transcranial Doppler Ultrasonography (TCDs) or by evidence of vasospasm on computerized tomography (CT) or catheter angiography. Multivariable logistic regression was performed to assess the relationship between polyuria and vasospasm.

**Results:**

95 patients were included in the study. 51 had cerebral vasospasm and 63 met the definition of polyuria. Patients with polyuria were significantly more likely to have vasospasm (OR 4.301, 95 % CI 1.378–13.419) in multivariate analysis. Polyuria was more common in younger patients (52 vs 68, *p* <.001) but did not impact mortality after controlling for age and disease severity. The timing of the development of polyuria was clustered around the diagnosis of vasospasm and patients with polyuria developed vasospasm faster than those without polyuria.

**Conclusions:**

Polyuria is common after aSAH and is significantly associated with cerebral vasospasm. The development of polyuria may be temporally related to the development of vasospasm. An increase in urine volume may be a useful clinical predictor of patients at risk for vasospasm.

## Background

Abnormalities of sodium and water excretion are frequently encountered after aneurysmal subarachnoid hemorrhage (aSAH) and include the cerebral salt wasting syndrome (CSW) and the syndrome of inappropriate antidiuretic hormone secretion (SIADH) [1, 2]. Natriuresis with polyuria is a common finding in aSAH and can contribute to hyponatremia [3]. Polyuria can also predispose to hypovolemia if urine losses are not replaced in a timely fashion. Even with attempts to replace urine losses, severe hypovolemia occurs in over half of aSAH patients [4]. Delayed cerebral ischemia (DCI) with or without cerebral infarction occurs in about 30 % of patients with aSAH and is strongly linked to cerebral vasospasm [5, 6]. Both the cerebral salt wasting syndrome and hyponatremia are associated with cerebral vasospasm [7]. In two studies, the onset of hyponatremia preceded the onset of cerebral vasospasm suggesting natriuresis may be a predictor of cerebral vasospasm [8, 9]. However, as hypovolemia in the setting of cerebral vasospasm can increase the risk of cerebral infarction, it is not known if CSW is an independent phenomenon or whether the concomitant hypovolemia explains the association [10]. Additionally, as current management of aSAH involves aggressive fluid therapy to maintain euvolemia and sodium replacement to maintain serum sodium levels, natriuresis often leads to polyuria without hyponatremia or hypovolemia. Anecdotally, clinicians involved in the treatment of aSAH have noted that an acute increase in urine output is a harbinger of cerebral vasospasm, irrespective of volume status. However, no studies have specifically examined the chronology of polyuria development and cerebral vasospasm risk.

The pathophysiology of polyuria after aSAH is not fully understood, but may be related to an increase in natriuretic peptides secondary to hypothalamic injury [11–13]. Importantly, it has been assumed that polyuria, when seen, leads to hyponatremia and hypovolemia, and these factors subsequently increases the risk of DCI and stroke. However, another explanation is that the CNS changes that lead to natriuresis after aSAH also lead to cerebral vasospasm. Thus, polyuria is related to the fundamental etiology of vasospasm and not simply a consequence of fluid replacement. In that case, polyuria may be able to identify patients at risk for vasospasm development independently of changes in volume status and hyponatremia. Given that urine output is a continuously measured, noninvasive parameter, the occurrence of polyuria may be useful for the early recognition of patients at high risk for cerebral vasospasm. We investigated whether the onset of polyuria is temporally associated with vasospasm.

## Methods

We performed a retrospective chart review of all patients admitted with aSAH to our institution between January 2012 and December 2013. The study was approved by the Hartford Hospital Institutional Review Board (IRB). Patients were included if they had an aneurysmal subarachnoid hemorrhage and were admitted within 48 h of aneurysmal rupture (i.e. onset of symptoms). In order to avoid the situation where patients develop polyuria, but die before the onset of vasospasm, patients were excluded if they died within 5 days of admission. Patients were also excluded if they received mannitol after the first 24 h of admission or had a history of or developed diabetes insipidus as these both are independent causes of polyuria. Finally, patients were also excluded if they developed a significant acute kidney injury (AKI) during their hospital course as this could mask the development of polyuria due to decreased glomerular filtration rate. We defined AKI as a 2-fold increase in serum creatinine as per both the RIFLE criteria and stage 2 in the Kidney Disease Improving Global Outcomes (KDIGO) grading system [14, 15].

Patients with aSAH were treated according to the current management guidelines including early aneurysm securing procedures, enteral nimodipine, and maintenance of a euvolemic state. All patients had frequent measurement of their intake and output and a negative fluid balance was avoided by intravenous fluid boluses and titration of intravenous fluid maintenance rates unless symptoms of hypervolemia indicated otherwise as per the treating the team. Central venous pressures as well as pulse contour analysis were utilized to ensure euvolemia if clinically indicated. Patients were regularly evaluated for DCI and cerebral vasospasm by frequent neurologic evaluation and Transcranial Doppler Ultrasonography (TCD). TCDs were performed at baseline (by post-bleed day 2) and then every 1–2 days (as indicated) until at least day 10. Patients suspected of having DCI or cerebral vasospasm were evaluated by computerized tomography (CT) or catheter angiography, and if present underwent hemodynamic augmentation therapy. Patients not responding to medical therapy underwent endovascular therapy including intra-arterial calcium channel blockers and balloon angioplasty.

Data were obtained from an IRB approved patient registry as well as the patients’ electronic medical records. 24 h urine output was obtained from the nursing flow sheets. Polyuria was defined as urine output >6 liters in a day. Cerebral vasospasm was defined on TCDs as a mean velocity >120 m/s plus a Lindegaard ratio >3 and on angiography or CT angiography based on the attending neuroradiologist’s interpretation. For consistency, any documentation of mild, moderate, or severe vasospasm was interpreted as positive for vasospasm however, terminology such as “minimal narrowing” was not. The onset day for cerebral vasospasm was the first day any test was consistent with cerebral vasospasm as defined above. Urine output was obtained between days 2 and 10 of admission. However, if cerebral vasospasm was diagnosed on or after day 10, urine outputs were recorded up to 24 h after the day of vasospasm onset. The onset of hyponatremia was also defined as the first day the serum sodium was below 135 meq/L excluding days 1 or 2 of admission. As the main objective of this study was to identify a temporal relationship with cerebral vasospasm, we excluded sodium or urine outputs on the first day of admission to eliminate pre-admission factors or factors related to early resuscitation efforts, surgical procedures or osmotic agents.

### Analysis

The polyuria group and non-polyuria groups were compared on demographics, comorbidities, and outcome. Then patients with vasospasm were compared to those without vasospasm by demographics, comorbidities, and outcome. Fisher score, Hunt Hess, and Glasgow Coma Scale (GCS) scores were dichotomized into mild and moderate/severe [16–18]. Many of the measures were categorical and dichotomized and were compared using Chi-square test. Continuous variables meeting assumptions of normality were analyzed using t-tests for independent groups. Wilcoxon Ranked Sum tests were substituted for ordinal variables (such as modified Rankin score) or any continuous variables not meeting the normality assumptions. All univariate tests were conducted two sided. Multivariable logistic regression was conducted for the prediction of vasospasm with polyuria as the key predictor of interest. Other predictors used included established risk factors for cerebral vasospasm such as Hunt Hess, GCS, and Fisher score as well as any other factors that showed a significant or near significant relationship to vasospasm or polyuria [19–21]. Statistical significance was defined as *p* <.05. Time to development of vasospasm was compared between those who developed polyuria prior to vasospasm (*n* = 42) and those who did not have polyuria (*n* = 32) using a log-rank test.

## Results

**Fig. 1:**
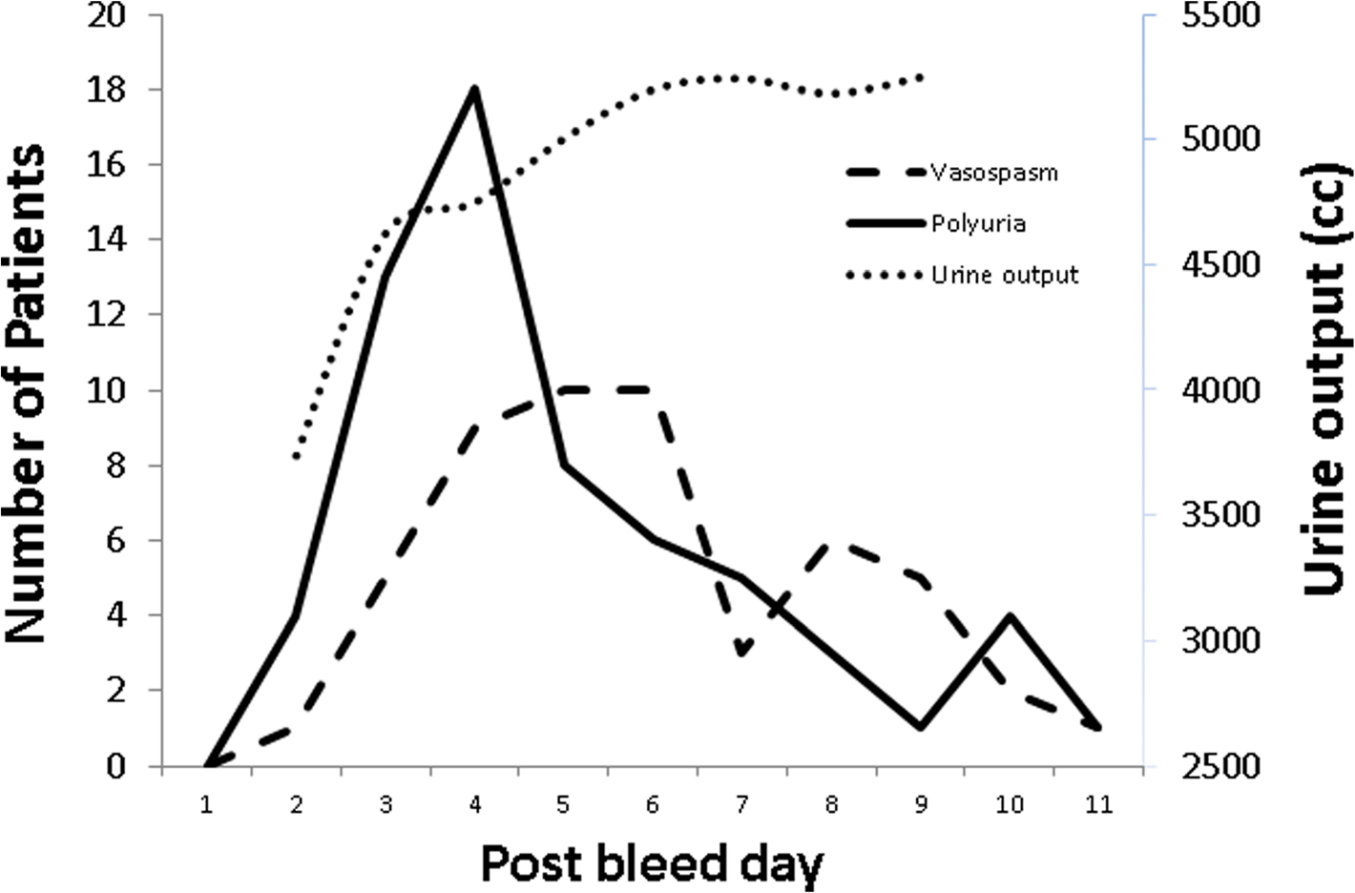
Vasospasm onset, polyuria onset, and average urine output by post-bleed day

107 patients with aneurysmal subarachnoid hemorrhage meeting inclusion criteria were identified. 10 patients were excluded secondary to death within 5 days of admission and 2 patients were excluded due to acute renal failure. Ninety five patients were included in the study. The mean age was 57.2 (range 24–91). 54 % (*n* = 51) of patients had cerebral vasospasm evident on at least one modality. More than half of these patients (57 %) were diagnosed between days 4 and 6 (see Fig. 1). Vasospasm was detected on TCDs only in 13 patients, CTA only in 19, angio only in 1 patient, and on TCD and CT or catheter angiography in 18 patients. Vasospasm was associated with low GCS, high Fisher score, high Hunt Hess score and the need for EVD placement (see Table 1).

**Table 1:** Baseline characteristics

	All Patients (*n* = 95)	Vasospasm (*n* = 51)	No Vasospasm (*n* = 44)	*P* value
Age, median ± SD	57	54 ± 10	61 ± 17	.**020**
Female	71.5	78.4	63.6	.171
GCS^1^ ≤ 13	38.9	49.0	27.2	.**036**
Fisher ≥ 3	75.8	86.3	63.6	.**016**
HH^2^ ≥ 3	51.6	62.7	38.6	.**024**
EVD^3^	70.5	84.3	54.5	.**003**
Polyuria	66.3	82.4	47.7	<.**001**

Glasgow coma score^1^ Hunt Hess^2^ External ventricular drain^3^. All values expressed as a percent unless otherwise indicated. Bold indicates a *p* value of less than 0.05 which is considered significant

Average daily urine output for the cohort is shown in Fig. 1. 66 % (*n* = 63) of patients had polyuria as defined by a 24 h urine output exceeding 6 liters on any day between days 2 and 10 of admission. These patients were significantly more likely to develop cerebral vasospasm in univariate analysis (*p* <0.01). Polyuria was significantly associated with younger age with a mean age of 52 in patients with polyuria vs an age of 66 in patients without polyuria (*p* <.001) (see Table 2). Polyuria was also associated with the use of hypertonic saline (*p* = .016) which was given to 27 of the 95 patients, 23 of whom developed polyuria. In multivariate analysis, after correcting for age, Hunt Hess Score, Fisher Grade, the use of hypertonic saline, and the need for ventriculostomy, patients with polyuria were 4 times more likely to develop cerebral vasospasm (OR 4.939, 95 % CI 1.511–16.142). Polyuria was not significantly associated with any other factor including Hunt Hess score, admission GCS, Fisher score, aneurysm location, sex, aneurysm securing procedure, or requirement for a ventriculostomy. There were no differences in mortality between those with polyuria and those without polyuria. 36 % (*n* = 34) of patients developed hyponatremia between days 3 and 10. The association between vasospasm and hyponatremia did not reach statistical significance.

**Table 2:** Baseline characteristics for patients with polyuria vs those without polyuria

	Polyuria (*n*=63)	No polyuria (*n*=32)	*P* value
Age, mean ± SD	52 ± 11	68 ± 14	<.**001**
Female	69.8	75.0	.639
Fisher ≥ 3	77.7	71.9	.614
HH^1^ ≥ 3	55.5	43.8	.288
EVD^2^	71.4	68.8	.815

Hunt Hess^1^ External ventricular drain^2^. All values expressed as a percent unless otherwise indicated. Bold value indicates *p* value of less than 0.05 which is considered significant

**Fig. 2:**
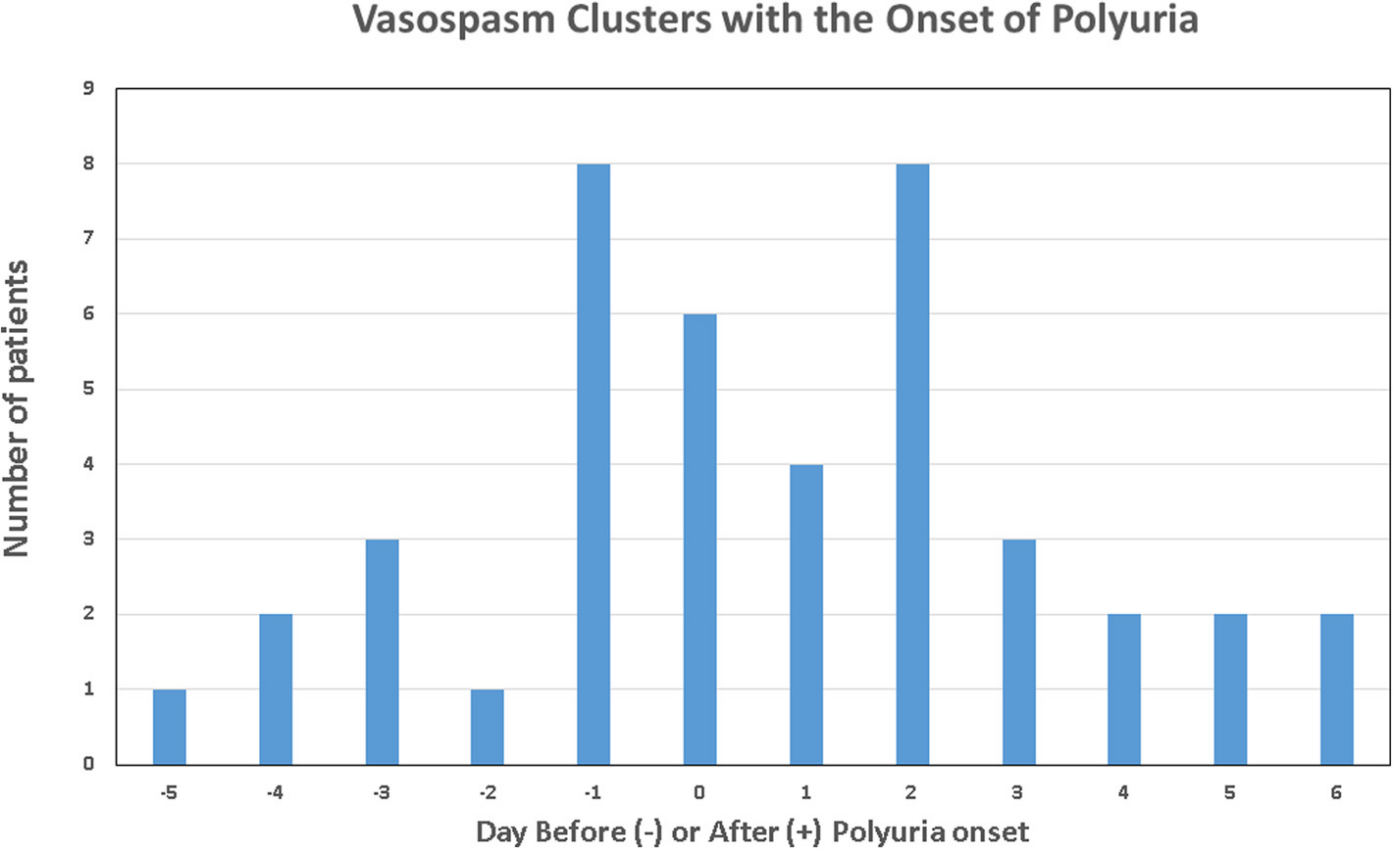
Vasospasm onset clusters around polyuria onset. Number of patients diagnosed with vasospasm in relationship to the day of polyuria onset. Day 0 is the day of polyuria onset

**Fig. 3:**
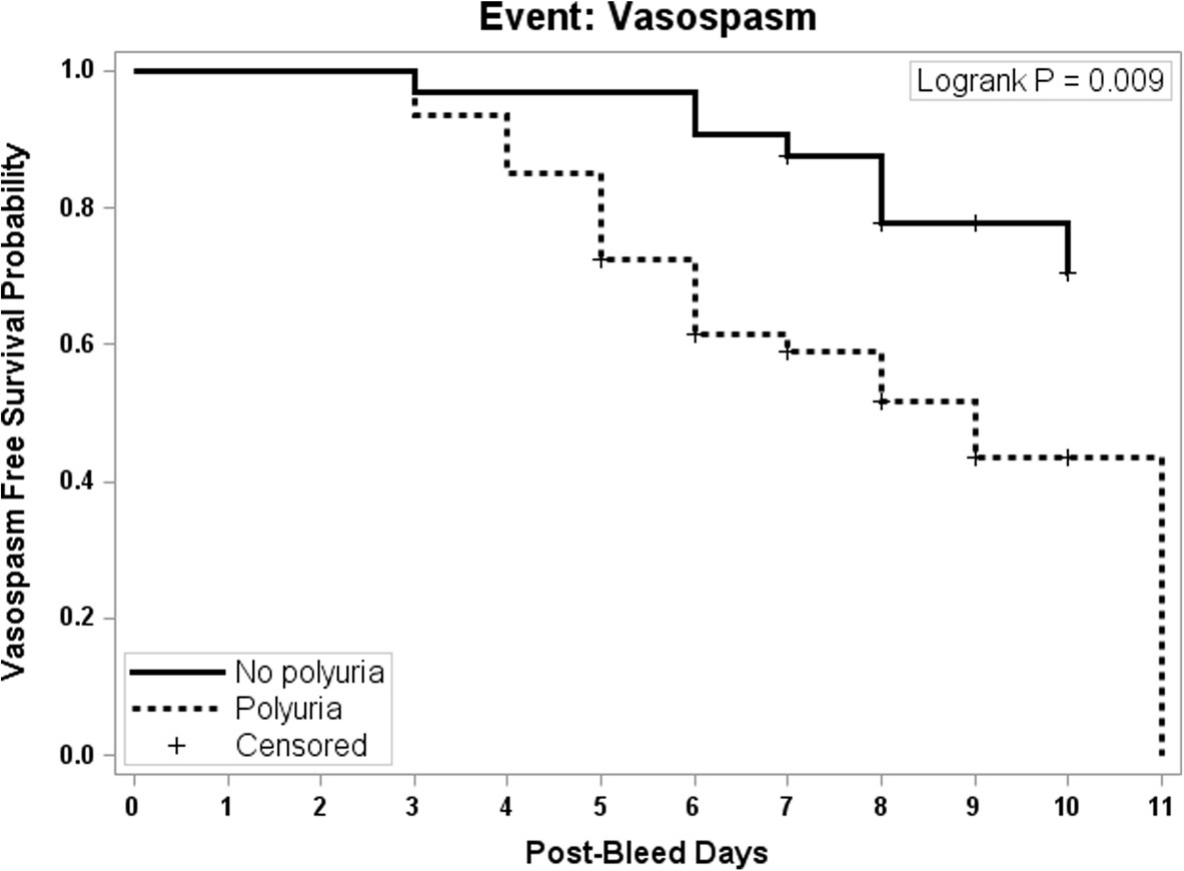
Kaplan-Meier Plot of time to vasospasm comparing the 42 patients with polyuria (excluding the 21 patients who developed vasospasm prior to developing polyuria) on the dashed line to the 32 patients without polyuria on the solid line

In all 95 patients, the onset of polyuria occurred earlier than documented cerebral vasospasm (see Fig. 1). In the 42 patients with polyuria and cerebral vasospasm, vasospasm detection was clustered around the time of polyuria onset (see Fig. 2). There were 43 patients who developed vasospasm after the onset of polyuria or in the absence of polyuria. Time-to-event analysis demonstrated that patients with polyuria developed vasospasm at a faster rate than those without polyuria (*p* = .0002, see Fig. 3).

## Discussion

In this study, we show that polyuria is common after aSAH and it is significantly associated with cerebral vasospasm. Polyuria tends to occur slightly earlier than cerebral vasospasm and the onset of vasospasm is clustered around the onset of polyuria. However, polyuria did not impact mortality after controlling for age and indicators of disease severity.

The incidence of vasospasm in this cohort is similar to prior studies [6, 22]. The incidence of polyuria was also high and this has not been previously reported. The majority of patients (66 %) developed polyuria on one specific day (excluding day 1) after aSAH. While we defined polyuria as a 24 h urine output above 6 liters, outside of the neurological intensive care unit polyuria has been relatively arbitrarily defined as a urine volume greater than 3 L within a 24 h period [23]. Using that definition would have been prodigiously liberal in our cohort and would have included 100 % of patients. Patients with aSAH are frequently maintained on intravenous fluids often in excess of 100 cc per hour. Given the additional enteral or oral intake, total fluid intake is rarely below 3 liters such that a urine output below 3 liters in the NICU is the exception rather than norm. Therefore, our definition of polyuria was more restrictive. However, our intention was not to show that a specific urine volume was associated with cerebral vasospasm, but rather to demonstrate the concept that an increase in or excessive volume of urine over baseline in any individual patient is a predictor of cerebral vasospasm. Based on our findings polyuria can serve as an easily obtainable clinical predictive factor to identify patients at risk for development of vasospasm.

Our study did not distinguish whether polyuria and vasospasm are independent phenomena that have similar etiologies or whether the onset of vasospasm contributes to polyuria or vice versa. The pathophysiology of excessive natriuresis and polyuria after aSAH is not completely understood however numerous studies have demonstrated an increase in natriuretic peptides including atrial natriuretic peptide (ANP) and brain natriuretic peptide (BNP) [12, 13, 24, 25]. Other less known peptides have been identified such as c-type natriuretic peptide and dendroaspis peptide [25, 26]. Hypothalamic dysfunction may be related to the severity of the SAH, both clinically and radiographically and could contribute to changes in the amount of these peptides released after SAH. A previous study found a higher incidence of hyponatremia with anterior communicating artery aneurysms and the putative mechanism was an increased incidence of hypothalamic dysfunction [27, 28]. We did not find an association between aneurysm location and the incidence of polyuria in this study. Regardless of the exact cause, the excessive secretion of natriuretic peptides could result in increased urinary sodium and volume loss leading to the clinical syndrome of cerebral salt wasting. However, there is considerable controversy regarding this pathophysiology. For example, the increase in natriuretic peptides may be a result of an associated cardiac injury which occurs in about 20 % of aSAH patients and is associated with vasospasm and DCI [29]. Importantly the association between polyuria and vasospasm held after controlling for well accepted measures of severity such as Hunt and Hess score, admission GCS and Fisher score in a multivariable model.

CSW is usually considered in the differential diagnosis of hyponatremia in aSAH patients, and is distinguished from SIADH by a clinical examination consistent with hypovolemia [30, 31]. However, as hypovolemia in aSAH patients is a known risk factor for cerebral infarction, aggressive measures are employed to prevent hypovolemia. Unlike SIADH in which isotonic fluids often exacerbate hypoanatremia, in CSW, replacement of renal losses with isotonic fluids may mitigate hyponatremia such that the only manifestation of this process is polyuria. Clinicians who frequently treat patients with aSAH are well-aware of sudden rises in urine output and anecdotally have associated this with cerebral vasospasm. While prior studies temporally linked hyponatremia with the onset of cerebral vasospasm, no study has tried to temporally link polyuria to cerebral vasospasm [8, 9]. Clinically, polyuria per se may be more useful than hyponatremia given that urine output is generally measured hourly in aSAH patients while sodium is usually measured daily. An increase in urine output may occur at any time during the day and may alert clinicians to the possibility of concomitant cerebral vasospasm. Furthermore, most centers try to avoid a negative fluid balance by titrating maintenance fluid rates in order to avoid hypovolemia. Therefore, making a diagnosis of cerebral salt wasting has become challenging for several reasons. First, hyponatremia is mitigated by the avoidance of a negative fluid balance and aggressive administration of saline. Second, even in patients who do develop hyponatremia, distinguishing CSW from SIADH is difficult given that the main distinguishing feature is volume status. Finally, even if attempts to avoid hypovolemia fail, as studies using pulse dye densitometry have shown, the bedside determination of volume status is often inaccurate [4, 32]. For these reasons, we believe that linking CSW to cerebral vasospasm is less clinically relevant than linking urine volume to vasospasm.

Our study is limited by its retrospective design. As stated above, it was not possible to completely rule out the possibility that some patients’ polyuria was a result of iatrogenic effects such as excessive fluid administration. While we excluded patients who received mannitol, we did not exclude patients who received hypertonic saline which could still have a mild diuretic effect although the relationship of polyuria to vasospasm persisted after controlling for this *in multivariate analysis*. Additionally, while urine output is a continuous parameter, the onset of cerebral vasospasm was limited to the timing of investigative tests. TCDs may not be performed daily in low risk patients without clinical changes and CTAs and conventional angiograms are often ordered only if vasospasm is suspected. Therefore, it is possible that vasospasm was missed in some patients or that the actual day of onset was earlier than recorded. It is also important to note that we chose to link polyuria to cerebral vasospasm rather than to delayed cerebral ischemia (DCI) which may be the more clinically relevant outcome than cerebral vasospasm [33]. This was not an oversight, but was intentional given the retrospective nature of this study, as linking polyuria to DCI would make it difficult to distinguish whether polyuria was a response to treatment. Once a patient is diagnosed with DCI, vasopressors are initiated and patients may receive additional fluid boluses, both of which may increase urine output. However, asymptomatic cerebral vasospasm is generally not treated with vasopressors or a change in fluid strategy. While our results may still be confounded by the patients who did develop DCI, by linking polyuria to mild vasospasm, which usually is not yet associated with ischemia, we believe the onset of polyuria coinciding with the onset of cerebral vasospasm was largely independent of iatrogenic issues. The retrospective design also made it difficult to determine who and when DCI was diagnosed as this is mainly an interpretation of chart notes whereas vasospasm was defined objectively by TCD changes and radiological findings. This study was also limited to the association with daily urine outputs. Future studies will examine hourly outputs and determine if precipitous changes in urine output are predictive of cerebral vasospasm. Future studies will also investigate whether the severity of vasospasm is associated with the incidence and degree of polyuria. Finally, while we did not detect an association of polyuria with mortality, clinical or radiological grade, or aneurysm location, a larger study may have detected an association as this initial study has limited power, despite being the largest to date on this topic.

## Conclusion

In summary, we have shown that polyuria is significantly associated with cerebral vasospasm and that polyuria onset is clustered with the onset of cerebral vasospasm. This may be useful in determining which patients are at high risk of developing vasospasm and developing future recommendations for the monitoring and disposition of these patients. A prospective study is necessary to further evaluate this association.

## Abbreviations

aSAH: (aneurysmal subarachnoid hemorrhage)
SIADH: (Syndrome of inappropriate antidiuretic hormone)
CSW: (Cerebral salt wasting)
DCI: (Delayed cerebral ischemia)
TCDs: (Transcranial Doppler Ultrasonography)
CT: (Computerized tomography)
IRB: (Institutional review board)
GCS: (Glasgow Coma Scale)
m/s: (meters per second)
cc: (cubic centimeters)
ANP: (Atrial natriuretic peptide)
BNP: (Brain natriuretic peptide)
